# Preparing for a hotter climate: A systematic review and meta-analysis of heatwaves and ambulance callouts in Australia

**DOI:** 10.1016/j.anzjph.2023.100115

**Published:** 2024-01-28

**Authors:** Mehak Oberai, Zhiwei Xu, Aaron J. E. Bach, Dung Phung, Jessica T. Watzek, Shannon Rutherford

**Affiliations:** 1 School of Medicine and Dentistry, Griffith University, Australia; 2 Cities Research Institute, Griffith University, Australia; 3 School of Public Health, The University of Queensland, Australia

**Keywords:** excess heat factor, ambulance services, heatwave action

## Abstract

**Objective:**

The objective of this study was to quantify the impact of heatwaves on likelihood of ambulance callouts for Australia.

**Methods:**

A systematic review and meta-analysis was conducted to retrieve and synthesise evidence published from 1 January 2011 to 31 May 2023 about the association between heatwaves and the likelihood of ambulance callouts in Australia. Different heatwave definitions were used ranging from excess heat factor to heatwave defined as a continuous period with temperatures above certain defined thresholds (which varied based on study locations).

**Results:**

We included nine papers which met the inclusion criteria for the review. Eight were eligible for the meta-analyses. The multilevel meta-analyses revealed that the likelihood of ambulance callouts for all causes and for cardiovascular diseases increased by 10% (95% confidence interval: 8%, 13%) and 5% (95% confidence interval: 1%, 3%), respectively, during heatwave days.

**Conclusions:**

Exposure to heatwaves is associated with an increased likelihood of ambulance callouts, and there is a dose–response association between heatwave severity and the likelihood of ambulance callouts.

**Implications for public health:**

The number of heatwave days are going to increase, and this will mean an increase in the likelihood of ambulance callouts, thereby, spotlighting the real burden that heatwaves place on our already stressed healthcare system. The findings of this study underscore the critical need for proactive measures, including the establishment of research initiatives and holistic heat health awareness campaigns, spanning from the individual and community levels to the healthcare system, in order to create a more resilient Australia in the face of heatwave-related challenges.

## Introduction

Recognised as one of the deadliest extreme weather events, heatwaves are prolonged periods of unusually high temperatures, which adversely impact human populations.^1^ For instance, in 2022, multiple heatwaves swept through the Northern Hemisphere leading to around 61,000 excessive deaths in Europe. Many more people across Asia and North America were severely impacted by the record-breaking high temperatures.^2,3^ Heatwaves have also resulted in an increased demand on healthcare systems, including ambulance services, emergency department (ED) visits, and hospital admissions across the globe.^4–6^ These have compounded the public health issue of ED overcrowding.^7,8^

In Australia too, heatwaves are becoming more frequent, intense, and longer lasting.^9–12^ This has led to significant human losses (354 heatwave-related deaths from 2002 to 2018 with almost two-thirds of the fatalities occurring during heatwaves of 2009 and 2014)^13^ and increased economic burden. For instance, during the summer seasons of 2014–2017, there were 1161 heatwave-attributable ED presentations with associated healthcare costs (thousands) of AUD $1020.3 in Adelaide.^14^ Also, as projected by Tong *et al*.,^15^ the heat-attributable costs are set to increase to AUD $125.8–129.1 million in the coming decades in Perth. This highlights the added historical and projected impacts of heatwaves on Australian healthcare systems.^16^

Ambulance services can play a key role in providing information about the health impacts of short-term exposure to environmental hazards such as heatwaves, given the real-time nature of ambulance attendances.^17^ By better understanding the magnitude of the impact of heatwaves on ambulance services, often the first entry point to the healthcare system for Australia, we can better project the added pressure due to heatwaves. The information provided can be useful to understand heat-prone areas through spatial and temporal mapping, leading to improved planning and preparedness for improving the allocation of services and resources to those in most need.^18–20^ Moreover, increased preparedness of ambulance services would help reduce the risk of health conditions proceeding to lethal conditions during hot weather.^13^ Hence, there is a need to recognise the effect of heatwaves on ambulance callouts for Australia to be better prepared and equipped for the warmer days.

Although the relationship between heatwaves and ambulance callouts has been explored for various Australian cities and states,^21–29^ there is a lack of an overall comprehensive quantitative summary of the association for Australia as a whole. This systematic review and meta-analysis was conducted to synthesise the available evidence about the association between exposure to heatwaves and the likelihood of ambulance callouts in the country.

## Methods

This review was conducted utilising the most up-to-date version of Preferred Reporting Items for Systematic Review and Meta-Analysis (PRISMA) guidelines.^30^ Studies were considered eligible for inclusion within this review if they met the criteria in Table 1.

Studies were required to have an epidemiological design with any experimental designs (i.e. randomised, and nonrandomised control trials) or reviews excluded. The initial literature search was conducted in 2021, and we wished to retrieve literature published in the past decade because we wanted to summarise the most recent evidence. We acknowledge that including all papers published from database inception to 2023 could have identified more papers.

As the main purpose of this review was to synthesise quantitative evidence on the impact of heatwaves on ambulance service use in Australia, we excluded studies relying exclusively on hospital, death registry, or ED admission records. Furthermore, as the primary exposure of interest is heatwaves, we excluded studies with air pollution as the primary exposure and temperature as a confounding exposure. We also excluded any study where the exposure was simply ‘*temperature*’ (and exposure not defined as ‘*heatwave*’) to ensure that data collection spanned the summer period of the respective location. Finally, we excluded studies where the performance or evaluation of ambulance and emergency services occurred without evaluation of the health impacts of heat. It should be noted that this study is an Australian extension of a systematic review and meta-analysis on global epidemiological evidence on heat and ambulance services usage (Xu *et al*.^31^). However, for this study, the inclusion/exclusion criteria were made more stringent.

### Information sources

A search strategy was developed and applied to six online databases: PubMed, Embase, Cumulative Index of Nursing and Allied Health Literature (CINHAL), Scopus, ProQuest, and Web of Science. The strategy was developed by the research team and was finalised with guidance and in consultation with a research librarian. A total of 38 MeSH or keywords and their combinations were searched using Boolean operators across three major themes: (i) a heat term, (ii) an ambulance term, and (iii) a health term (see Supplementary 1 for full search strategy)”. The final search was conducted on 31^st^ May 2023.

### Screening, data extraction and quality assessment

All the references acquired from the systematic search of the databases were imported into Endnote (version X9, 2013), and duplicates were removed before uploading the references to Covidence (v2715, 2021) to complete screening, data extraction and quality assessments.

Two independent reviewers (MO and ZX, AB, or JW) screened the full texts using established inclusion/exclusion criteria. Any conflicts were resolved via discussion between both reviewers, and if consensus could not be reached, a third reviewer (SR or DP) was sought. A single reviewer (MO) performed the data extraction for the papers which passed the full-text screening. Data extracted from papers meeting all inclusion criteria included general information (author, year, study location [state, cities, and regions]), methodological qualities (study design, subgroup populations, exposure(s), heat measure, adjusted variable(s), health outcome, ambulance/emergency service call-out reasons/cause (s), effect measure, reference group for measuring effect, effect size), and a narrative summary of main results, study strengths, and limitations.

Two reviewers (MO, AB or ZX) conducted quality assessment analyses using a tool adapted from the Newcastle–Ottawa Scale (NOS) for assessing the quality of nonrandomised studies.^32^ The tool was adapted to suit each of the included study designs (time-series, cross-sectional, cohort, case-control and case-series, and case-crossover). Quality assessment analyses included examining sample representativeness, ascertainment of exposure and outcome measures, inclusion of common confounders within the statistical models used, and the specificity of the outcome presented (Supplementary 2).

### Data synthesis and analysis

The included studies used different effect estimate indicators to report the association between heatwaves and ambulance callouts, including incidence rate ratio (IRR), relative risk (RR), and odds ratio (OR). We assumed that OR was a reasonable approximation of RR in the eligible studies because ambulance dispatches are small probability events.^33^ We were unable to convert IRR into RR because the published papers did not provide the raw data for IRR calculation. Hence, we presented results for both the pooled IRR and pooled RR in the results section. This is the best practice to not pool IRR and RR together although both are measures of association.

The eight eligible studies for inclusion were meta-analysed into one of the three groups i.e. (i) heatwaves and ambulance callouts for all causes, (ii) heatwaves and ambulance callouts for cardiovascular causes, and (iii) heatwave intensity and ambulance call outs for all causes (Table 2). Although only a total of eight studies were included, four studies had different subgroups which were treated as separate study estimates for this analysis.^34^ This is the case of “multilevel meta-analysis” where a three-level model is being used. When effect sizes are dependent, because they are based on the same sample or are obtained from the same set of researchers, it is unsuitable to use the standard two-level models because they assume conditional independence of the effect sizes, meaning that no relationships among effect sizes exist once controlling for the moderators in the model. In such situations, a three-level model might be adopted to accommodate the dependency.^35^

Further multilevel (hierarchical) meta-analysis or random-effect meta-analysis was carried out using restricted maximum likelihood (REML) method to quantify the effects of heatwaves on ambulance callouts. The heterogeneity was assessed using Cochran’s Q and was categorised as low (≤25%), moderate (26–74%), or high (≥ 75%), using I^2^ statistics. Though all selected studies did not have the same definitions of heatwaves (exposure), they were pooled together to advance our collective knowledge in order to raise awareness of heat health impacts and identify the need for more uniform methodologies for future heat health impact research.

All the statistical analyses were carried out using the R software (version 4.2.3) using “*meta*” and “*metafor*” package.

## Results

### Study selection

**Figure 1:**
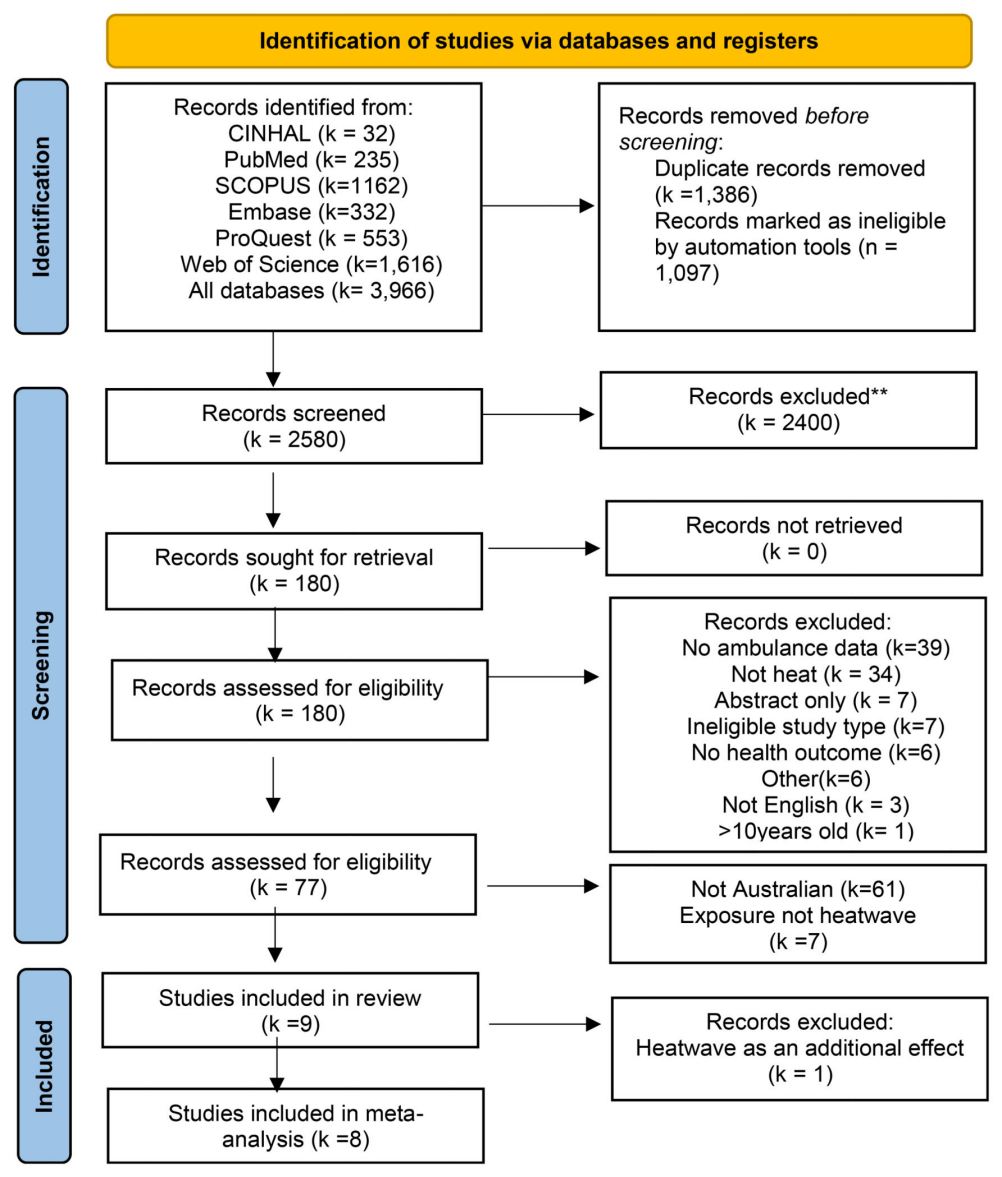
PRIMSA for literature search. PRIMSA = Preferred Reporting Items for Systematic Review and Meta-Analysis.

The PRISMA diagram in Figure 1 describes the process utilised for record selection for both the initial database searches and bibliographic screening. Overall, 77 records were identified during the global epidemiological review. Following screening for Australian studies, this number was reduced to 16 studies for the time period between 1^st^ Jan 2011 and 31^st^ May 2023. Of these 16 records, only nine studies met the inclusion criteria for the review, and eight of the nine studies have been included in the meta-analysis. Turner *et al*.^21^ examined the main and added effects of heatwaves rather than the overall effect of heatwaves; therefore, it was excluded from the meta-analysis. The added effect is the effect of ‘waves’ but not ‘heat’ per se.

### Study characteristics

Table 3 displays the individual study characteristics and methodological parameters for all the nine studies. Queensland has the highest number of studies (k = 3),^21–23^ followed by South Australia (k = 2),^24,25^ and New South Wales (k = 2),^26,27^ whereas Western Australia (k = 1)^28^ and Tasmania (k = 1)^29^ had one study conducted.

A wide range of heatwave definitions have been used in the included studies. Some used excess heat factor (EHF), whereas a few combined threshold temperatures and duration to define heatwaves. EHF is an index based on a three-day-averaged daily mean temperature and is intended to capture heatwave intensity.^36^ This index supports an intensity and classification scheme which is relative to the local climate. Seven^21,22,24–28^ of the selected studies included ambulance callouts for all causes, whereas only five^21,23,24,28,29^ specifically reported the ambulance callouts for cause-specific health conditions including cardiovascular, neurological, and respiratory causes. In all the studies (k = 9), the comparator was no-heatwave day.

### Quality assessment

Table 4 displays the quality assessment of all the studies based on the study design, using the adapted NOS tool.^32^ One (Campbell *et al*.^29^) of the studies was of high quality (case-crossover design), whereas the other eight were of moderate quality (two case-crossover^23,24^ and five^21,22,25–28^ time-series design).

### Meta-analysis

**Figure 2:**
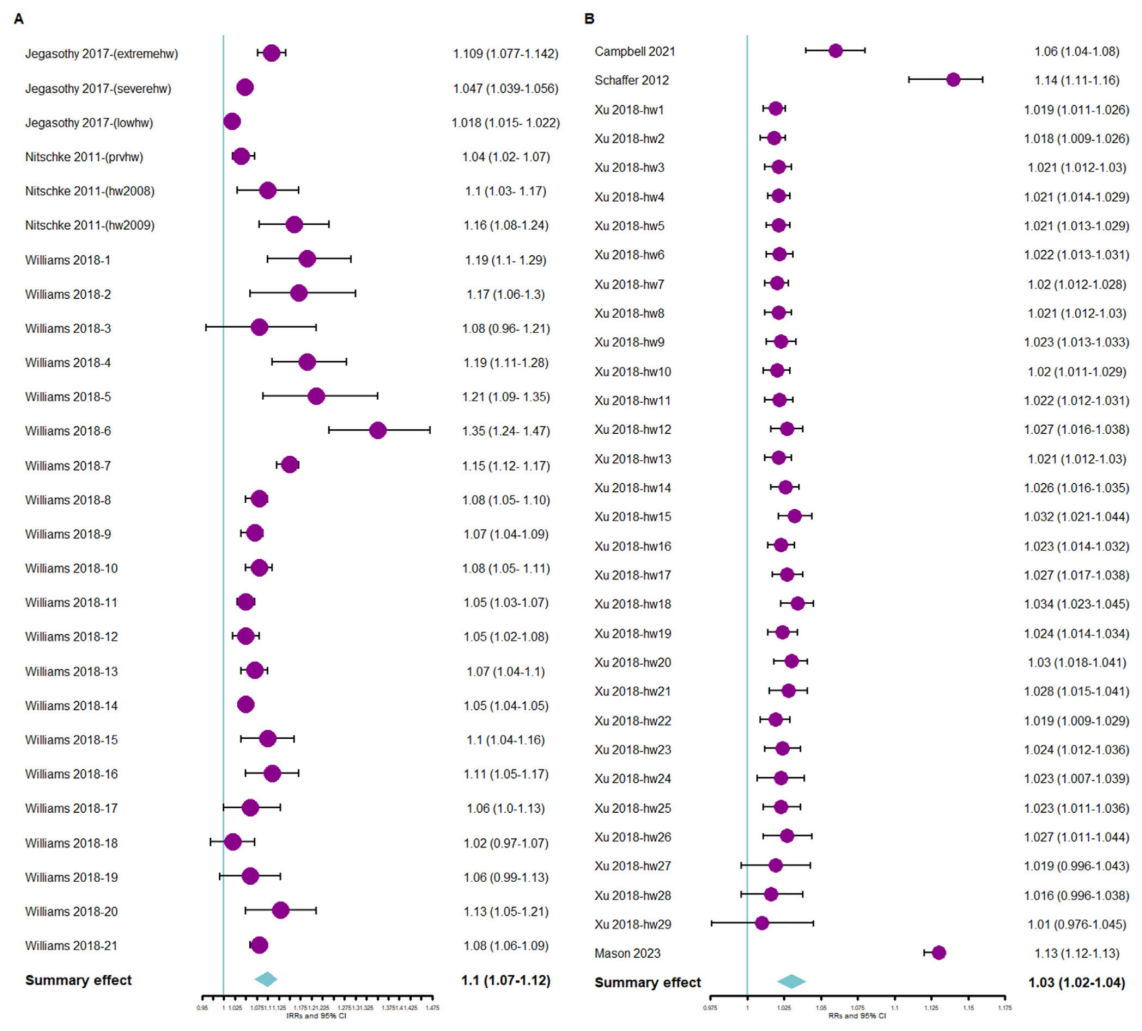
(a) Forest plot showing association between ambulance callouts and heatwaves using pooled IRR and (b) Forest plot showing association between ambulance callouts and heatwaves using pooled RR. Abbreviations: IRR = incidence rate ratio; RR = relative risk.

The pooled IRR showed that the rate of people requiring ambulance services during heatwave days was 10% (95% confidence interval: 7–12%) higher than that during non-heatwave days (Figure 2A). The pooled RR showed that the risk of ambulance dispatches increased by 3% during heatwave days as compared to that during non-heatwave days (Figure 2B).

**Figure 3:**
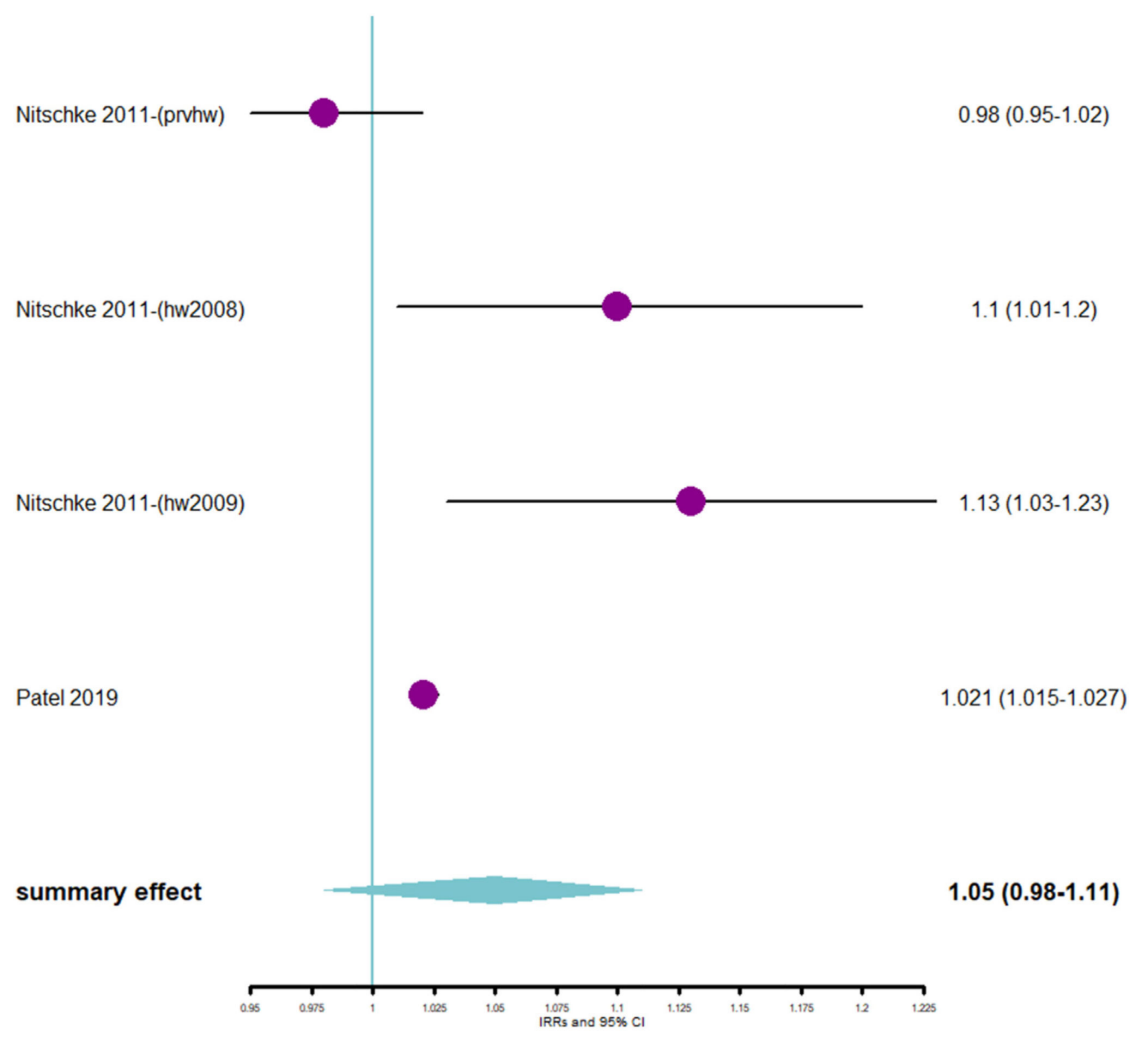
(a) Forest plot showing association between ambulance callouts due to cardiovascular causes and heatwaves using pooled IRR. Abbreviation: IRR = incidence rate ratio.

**Figure 4:**
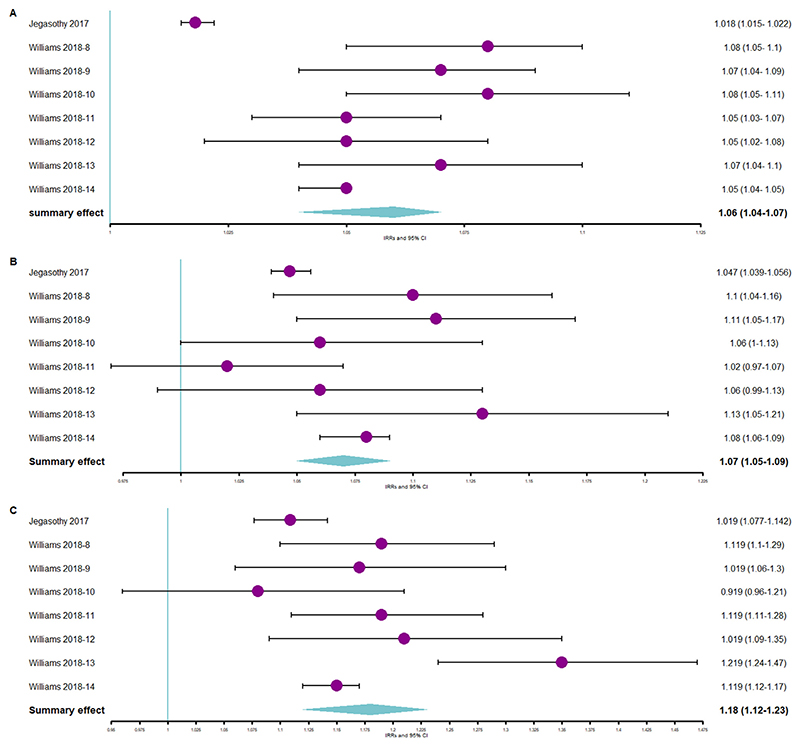
(a) Forest plot showing association between ambulance callouts and low-intensity heatwaves using pooled IRR. (b) Forest plot showing association between ambulance callouts and severe-intensity heatwaves using pooled IRR. (c) Forest plot showing association between ambulance callouts and extreme-intensity heatwaves using pooled IRR. Abbreviation: IRR = incidence rate ratio.

Compared with non-heatwave days, ambulance callout rates increased by 5% for cardiovascular causes (Figure 3) during heatwave days. Compared with non-heatwave days, the all-cause ambulance callout rate increased by 6% (Figure 4A), 7% (Figure 4B), and 18% (Figure 4C) during low-intensity, severe, and extreme heatwave days, respectively. Additionally, the pooled RR showed that risk of ambulance callout increased by 13%, 13%, and 15% (Figure S1.A, S1.5B, AND S1.C, respectively, in supplementary 3) during low-intensity, severe, and extreme heatwave days, respectively, when compared with non-heatwave days.

There was high heterogeneity for all the meta-analyses (I^2^ ≥ 75% in each case) of this study, and lower number of studies limited the possibility to calculate the publication bias.^37^

## Discussion

This is the first review to consolidate the evidence relating to the impact of heatwaves on the likelihood of ambulance callouts across Australia. The identified association for an increased rate of ambulance callouts associated with heatwave exposure across Australia is similar to the increase in demand for other healthcare services including ED visits and hospital admissions during the heatwave period,^17,38–41^ both in Australia and worldwide, underlining the increased pressure on healthcare services during heatwave periods.

The ageing population, growing prevalence of chronic diseases, and increased incidence of other extreme weather events in the country due to climate change is already pushing the healthcare system to its capacity. Australia has been experiencing droughts, bushfires, and floods over the past few decades, and with Covid-19 pandemic, the system is facing significant issues of workforce and infrastructure shortages and staff fatigue.^42^ Extreme events are set to be costly—an economic analysis calculated the healthcare costs associated with bushfires between 2021 and 2030 to be around AUD $69 million.^43^ Occupational illnesses and injuries due to heatwaves are projected to cost around AUD $4.3 million, annually.^43^ This does suggest that the increased projection for heatwaves for Australia has the potential to add on to the already at-capacity Australian healthcare system.

### Intensity of heatwaves and causes for ambulance callouts

The pooled estimate showed that the elevation in the likelihood of ambulance callouts was the greatest for extreme heatwaves, highlighting that heatwave intensity is an important factor when determining the health impact of heatwaves.

Similar to the findings of a review and meta-analysis conducted by Liu *et al*.,^44^ where mortality and morbidity increased during the heatwaves, and by Zang *et al*.,^45^ where it was found that heat extremes adversely affect people suffering from cardiovascular diseases, this analysis identified that the ambulance callout rate increased by 5% for cardiovascular causes on heatwave days compared to non-heatwave days for Australia. Findings of a scoping review conducted by Gestal Romani *et al*.^46^ also support these findings. Findings of Campbell *et al*.^29^ and Nitschke *et al*.^24^ reveal some interesting results where the ambulance call out rate increased for respiratory and neurological causes. However, data for these and other heat-sensitive health conditions were not available for carrying out meta-analysis.

Given we know that multiple health conditions such as cerebrovascular, respiratory, endocrine, and genitourinary conditions can be exacerbated by extreme heat,^5^ this highlights a major research gap. The main reason for this gap is the lack of cause-specific ambulance callout data. Data linkage can be a solution to fill in this gap. For instance, linking patients’ ambulance-callout data with their hospitalisation or ED visit data could help in determining the cause of ambulance callout.^31^

### Population subgroups

There were not enough studies to identify the pooled effect of the heatwaves on ambulance services for particular groups such as those aged over 65 years or young children, who are more vulnerable to heat than are other populations.^47,48^ Though population subgroups existed in a few studies, it was not possible to pool them together because of inconsistent definition of population groups. For instance, Campbell *et al*.^29^ defined older people as those aged >65 years (excluding 65) and Nitschke *et al*.^24^ defined this group including persons aged 65 years. Similar disparities were also seen for other age groups.

### Limitations

The major limitation of this review is that studies were pooled together even though the definition of the exposure was not uniform. For instance, Campbell *et al*.^29^ used the Australian Meteorology Office’s EHF definition. On the other hand, Nitschke *et al*. 2011^24^ defined heat waves as daily maximum temperature (Tmax) being ≥35 °C (95th centile) for three or more consecutive days. This perhaps reflects the lack of consistent heatwave definition based on EHF for Australia prior to 2014 due to varied climate zones.^36^ Another limitation which impacted the clear interpretation of the results was use of different outcome measures across studies and lack of availability of raw data to calculate a uniform measure. This resulted in pooling of the available limited data into two separate pools based on measure of association. Over-reliance on a few studies is another limitation that must be accounted for as this might have impacted the pooled estimate. Moreover, the limited number of studies in each group being less than ten limited our capacity to look for publication bias and determine the robustness of our results. Another limitation of the study is that it used the NOS tool for assessing the risk of bias, which was considered the gold standard at the time of the study’s execution. Nevertheless, it is worth acknowledging that the landscape of systematic reviews for environmental exposures has evolved, and the field now increasingly relies on the Navigation Guide framework for evaluating study quality and the risk of bias.^44,49^

### Recommendations

To overcome some of the limitations, further research and analysis should involve usage of a standard definition for heatwave (EHF-based) and more group-specific (gender, age, socio-economic status, heat-sensitive health conditions) exploration of the impact of heatwaves on ambulance services. The current study recommends using statistical methods of association, which could make it easy to calculate a comprehensive quantifiable outcome.

Linking spatial data with ambulance data should be considered to identify specific heat-prone areas in order to reduce health burden via improved prevention. This can also aid in better local surveillance of heat-related morbidities and mortalities, particularly as more heat prevention interventions are implemented. Factors such as heterogeneous climate zones and humidity of a location should also be accounted along with EHF, during the spatial mapping of heat-prone areas along with the socio-demographic and economic elements. This would also help in understanding the distribution of heatwave risk, while contributing to overall understanding of the heatwave vulnerability of the Australian population.^50,51^

This study adds to the growing evidence that ambulance-callout likelihood increases during a heatwave. Thus, besides carrying out further research and improved planning to prepare our healthcare system to tackle this situation, the findings draw attention to the need for increased preparedness and awareness around heatwaves in individuals and the community. Heat action plans, and focussed heat-risk-reduction policies need to consider individual, interpersonal, and community factors as all these factors collectively impact the sensitivity and adaptive capacity of individuals and communities.^52^ In conjunction with this, should individuals start undertaking preventative measures at the levels of community, individual, and interpersonal interactions, it becomes feasible to anticipate a reduction in the adverse consequences associated with heatwaves^53^ and reduce the demand of ambulance callouts that this meta-analysis highlights.

## Conclusion

The evidence presented via this meta-analysis suggests a statistically significant increase in the likelihood of ambulance callouts across Australia during heatwave events. The rate and risk of ambulance callouts increased with increasing intensity of heatwaves. Coinciding with the average temperatures projected to rise even further in future; the associated frequency, intensity, and duration of heatwave events will present ever-increasing health service demands on an already stressed healthcare system. This foresight reinforces the urgent need for the whole of health service, including ambulance services’ capacity building and heat preparedness planning at all levels.

## Supplementary Material

**Appendix A Supplementary data**

Supplementary data to this article can be found online at https://doi.org/10.1016/j.anzjph.2023.100115.

Supplementary file

## Figures and Tables

**Table 1:** Inclusion criteria.

Inclusion	
Participants/population	The research was restricted to human populations.
Intervention/exposure	Included a measure of heatwave as the primary exposure.
Comparator	Heatwave (different heatwave definitions ranging from excess heat factor [EHF] to others) versus no heatwave
Outcome	Used routinely collected ambulance records to investigate outcomes, and the outcome was public health–related (e.g. not performance assessment of ambulance services, nor occupational based).
Language	Studies published in English
Timeline	Studies published between 1^st^ Jan 2011 and 31^st^ May 2023
Study design	Epidemiological design
Publication status	Peer-reviewed published articles
Location	Australia

**Table 2:** Groups for data analysis.

Group id	Description		No. of studies	No. of effect estimates	Studies
**1**	**Heatwaves and ambulance callouts for all causes**				
1.1	Heatwave event: total (RR)	heatwave versus non-heatwave; all causes	4	31	[21, 22, 26, 29]
1.2	Heatwave event: total (IRR)	heatwave vs. non-heatwave; all causes	3	27	[24, 25, 27]
**2**	**Heatwaves and ambulance callouts for cardiovascular causes**				
2.1	Heatwave event: cardiovascular (IRR)	heatwave versus non-heatwave; cardiovascular diseases	2	4	[24, 28]
**3**	**Heatwave intensity and ambulance callouts for all causes**				
3.1.1	Extreme-intensity heatwave (RR)	extreme heatwave versus non-heatwave; all causes	2	2	[25, 27]
3.1.2	Extreme-intensity heatwave (IRR)	extreme heatwave versus non-heatwave; all causes	2	8	[25, 27]
3.2.1	Moderate-intensity heatwave (RR)	moderate heatwave versus non-heatwave; all causes	2	2	[25, 27]
3.2.2	Moderate-intensity heatwave (IRR)	moderate heatwave versus non-heatwave; all causes	2	8	[23, 29]
3.3.1	Low-intensity heatwave (RR)	low heatwave versus non-heatwave; all causes	2	2	[23, 29]
3.3.2	Low-intensity heatwave (IRR)	low heatwave versus non-heatwave; all causes	2	8	[23, 29]

Abbreviations: IRR = incidence rate ratio; RR = relative risk.

**Table 3:** Study characteristics (included).

First Author	States examined	Cities examined	Study design	Subgroup population	Measure of heat (Exposure)	Outcome	Cause(s)	Measures of effect	Reference Group for Measure of Effect
Turner 2013	QLD	Brisbane	Time series	All, age groups	Heatwave as an additional effect	Ambulance attendance	All causes, cardiovascular, respiratory	% Increase in ambulance attendance	No heatwave
Xu 2018	QLD	Brisbane	Time series	All ages	Different heatwave definitions	Ambulance callouts	All causes	RR	Comparing the RR for different definitions used in the study
Mason 2023	QLD	Major cities, inner region, outer region, remote, and very remote	Case crossover	All ages	Heatwave (defined using EHF)	Ambulance callouts	Cardiovascular, respiratory, specified medical, obstetric, injuries and heat and cold exposures based on MPDS categories	RR	No heatwave
Nitschke 2011	SA	Adelaide	Case series	All age groups	Heatwave in 2008 and 2009	Ambulance callout	All causes, cardiac, respiratory, neurological	IRR	Previous heatwave
Williams 2018	SA	Adelaide	Time series	Regional population groups	Heatwave (defined using EHF)	Ambulance callouts	All causes	IRR	No heatwave
Jegasothy 2017	NSW	Major cities, inner region, outer region, remote, and very remote	Time series	Major cities, inner regional, outer regional, whole of NSW	Heatwave (defined using EHF)	Ambulance callout	All causes	IRR	No heatwave
Schaffer 2012	NSW	Sydney	Time series	All ages, age groups	Heatwave	Ambulance callout	All causes	RR	No heatwave
Patel 2019	WA	Perth	Time series	Age groups, gender, socio-economic status	Heatwave (defined using EHF)	Ambulance callout	All causes, cardiac issues, dehydration, endocrine issues	IRR	No heatwave
Campbell 2021	TAS	All regions	Time- stratified case&shy;crossover	All, age groups, socio-economic status	Heatwave (defined using EHF)	Ambulance dispatch	Cardiovascular, respiratory, renal, diabetic, psychological, direct heat-related and other heat-related conditions	OR	No Heatwave

* QLD = Queensland; SA = South Australia; NSW = New South Wales; WA = Western Australia; TAS = Tasmania; EHF = excessive heat factor; MPDS = Medical Priority Dispatch System; RR = relative risk; IRR = incidence rate ratio; OR = odds ratio.

**Table 4:** Quality assessment results.

Case- crossover design					
Study	Domain				
	Selection	Exposure	Comparability		Overall
Campbell 2021	Mod	High	High		High
Nitschke 2011	Mod	Mod	High		Mod
Mason 2023	Mod	High	Mod		Mod

Time series design					
**Reference**	**Domain**				
	**Selection**	**Exposure**	**Comparability**	**Outcome**	**Overall**
Jegasothy 2017	High	Mod	High	Low	Mod
Patel 2019	High	Mod	High	Low	Mod
Schaffer 2012	High	Mod	Mod	High	Mod
Turner 2013	High	Mod	High	Low	Mod
Williams 2018	High	Mod	Mod	Low	Mod
Xu 2018	High	Mod	Mod	Low	Mod
