# Supplementary material for: Preparing for a hotter climate: A systematic review and meta-analysis of heatwaves and ambulance callouts in Australia
Source: Aust N Z J Public Health. Author manuscript; Available in PMC 2026 Jan 6. (PMC7618567; doi:10.1016/j.anzjph.2023.100115)

**Supplementary Materials**

**Ambulance call outs and heatwaves in Australia: A systematic review and metanalysis**

**Supplementary 1. Complete Seach Strategy**

| *Heat terms*  ("heat" OR "heat wave" OR "heatwave" OR "hot day" OR "hot weather" OR "hot temperature" OR "extreme heat" OR "extreme hot day" OR "temperature" OR "hot temperature" OR "high temperature" OR "season" OR "seasonal" OR "seasonal variation" OR "summer" OR "warm weather" OR "warm day" OR "extreme environment") |
| --- |
| AND |
| *Ambulance terms*  ("ambulance" OR "emergency service" OR "emergency medical technician" OR "paramedic" OR "emergency medical service" OR "ambulance call out" OR "ambulance dispatch" OR "emergency health service" OR "emergency medical treatment" OR "pre-hospital" OR "prehospital" OR "ambulance call out") |
| AND |
| *Health terms*  ("health" OR "morbidity" OR "mortality" OR "comorbidity" OR "health condition" OR "death" OR "disease" OR "illness") |

**Supplementary 2. Quality Assessment Tools**

Time-series design quality assessment tool

| Question | Responses |
| --- | --- |
| Selection | |
| Representativeness of the sample | |
|  | Truly representative of the target geographical area (entire area or random sample)*** |
|  | Somewhat representative of the target geographical area (non-random sampling)** |
|  | High likelihood of being unrepresentative of the target geographical area (e.g., convenience sample)* |
|  | No description of the derivation of the sample |
| Exposure | |
| Ascertainment of ambient temperature exposures defined by the authors (e.g., defined by temperature thresholds, or by geographic regions) | |
|  | Secure instrumental record (e.g., thermometers in weather stations, satellite data, established heatwave/cold bell)*** |
|  | Structured interview** |
|  | Written self-report* |
|  | No description |
| The size of the regional unit in the temperature exposure assignment | |
|  | Smaller than (not including) city in the U.S., county in China, or the equivalent in other countries; smaller than 15km (not including) in grid side length regarding grid temperature exposures; or smaller than (not including) 8.5km regarding distance from temperature monitors*** |
|  | Equal to city in the U.S., county in China, or the equivalent in other countries; ranging from 15km (including) to 35km (including) in grid side length regarding grid temperature exposures; or ranging from 8.5km (including) to 20km (including) regarding distance from temperature monitors** |
|  | Larger than (not including) city in the U.S., county in China, or the equivalent in other countries; larger than (not including) 35km in grid side length regarding grid temperature exposures; or larger than (not including) 20km regarding distance from temperature monitors* |
|  | No description or the regional unit is unknown |
| Comparability | |
| The study controls for long-term trends, seasonality, and day of week (when the outcome is daily). | |
|  | Yes* |
|  | No |
| The study controls for at least one other important potential confounder (air pollutants). | |
|  | Yes* |
|  | No |
| Outcome | |
| Assessment/specificity of outcome | |
|  | Ambulance data limited to heat related codes only AND accidental causes removed*** |
|  | Ambulance data limited to heat related codes only OR accidental causes removed** |
|  | All causes followed, or specific causes (e.g. CVD, stroke)* |
|  | No description of specificity |
| Quality assessment | |
| Overall score (out of 14 stars) | |
|  | Low 0-6 stars (0-49%) |
|  | Moderate 7-11 stars (50-84%) |
|  | High 12-14 stars (85-100%) |
| Comments: |  |

*- one star; **- 2 stars; ***- 3 stars

Case-crossover design quality assessment tool

| Question | Responses |
| --- | --- |
| Selection | |
| 1. Is the case definition adequate? | |
|  | Yes, with independent validation (e.g., >1 person/record/time/process to extract information, or reference to primary record sources such as insurance records, medical/hospital records, or death certificates)** |
|  | Yes, e.g., record linkage or based on self-reports, with no reference to primary record* |
|  | No description |
| 2. Representativeness of the cases | |
|  | Consecutive or obviously representative series of cases (e.g., all eligible cases with outcome of interest over a defined period of time, all cases in a defined catchment area, all cases in a defined hospital or clinic, or an appropriate sample of those cases [e.g., random sample]) * |
|  | Potential for selection biases (i.e., does not satisfy requirement in part (a) or not stated) |
| 3. Selection of controls | |
|  | Community controls (e.g., same source population as cases and would be cases if had outcome; representative of the source population [e.g., a random sample of the source population])** |
|  | Hospital controls (or other controls that are not representative of the source population)* |
|  | No description |
| 4. Definition of controls | |
|  | No history of disease outcome (if cases have new [not necessarily first] occurrence of outcome, then controls with previous occurrences of outcome of interest should not be excluded)* |
|  | No mention of history of disease outcome |
| Comparability | |
| 5. The study controls for at least one other important potential confounder (air pollutants). | |
|  | Yes* |
|  | No |
| Exposure | |
| 6. Ascertainment of ambient temperature exposures defined by the authors (e.g., defined by temperature thresholds, or by geographic regions) | |
|  | Secure instrumental record (e.g., thermometers in weather stations, satellite data, established heat waves/cold spells)**** |
|  | Structured interview where blind to case/control status*** |
|  | Interview where not blind to case control status** |
|  | Written self-report* |
|  | No description |
| 7. Same method of ascertainment of ambient temperature exposures for cases and controls. | |
|  | Yes* |
|  | No |
| 8. The size of the regional unit in the temperature exposure assignment. | |
|  | Smaller than (not including) city in the U.S., county in China, or the equivalent in other countries; smaller than 15km (not including) in grid side length regarding grid temperature exposures; or smaller than (not including) 8.5km regarding distance from temperature monitors*** |
|  | Equal to city in the U.S., county in China, or the equivalent in other countries; ranging from 15km (including) to 35km (including) in grid side length regarding grid temperature exposures; or ranging from 8.5km (including) to 20km (including) regarding distance from temperature monitors** |
|  | Larger than (not including) city in the U.S., county in China, or the equivalent in other countries; larger than (not including) 35km in grid side length regarding grid temperature exposures; or larger than (not including) 20km regarding distance from temperature monitors* |
|  | No description or the regional unit is unknown |
| Quality Assessment | |
| Overall score (out of 15 stars) | |
|  | Low 0-7 (0-49%) |
|  | Moderate 8-12 (50-84%) |
|  | High 13-15 (85-100%) |
| Comments: |  |

*- one star; **- 2 stars; ***- 3 stars

**Supplementary 3. Figure S1 (A: Pooled RR for low-intensity heatwave; B: Pooled RR for severe-intensity heatwave; C: Pooled RR for extreme-intensity heatwave)**


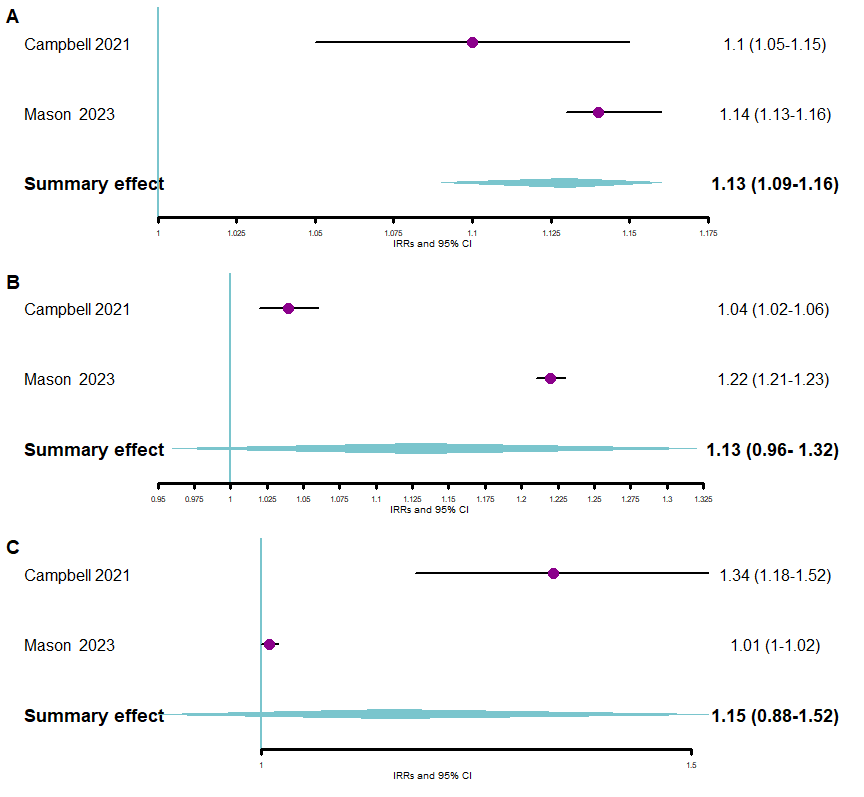

Supplement: Supplementary file [file EMS211695-supplement-Supplementary_file.docx]
